# Supplementary material for: Health Interventions for the Prevention of Dehydration in Agricultural Workers Exposed to Heat Stress: A Systematic Review
Source: Healthcare (Basel). 2025 May 23;13(11):1232. doi: 10.3390/healthcare13111232 (PMC12155329; doi:10.3390/healthcare13111232)
Supplement: Supplementary file 1 [file healthcare-13-01232-s001.zip › Supplementary Material S5.pdf]

HANDLING MISSING DATA

In this systematic review, missing data were carefully and systematically managed at various stages of the review process to maintain the rigor and comprehensiveness of the analysis.

During the Initial Selection (n=558): These articles were retained for full-text review if other available information suggested relevance to our research question on dehydration and heat stress in agricultural workers. The articles were individually reviewed to determine the publication language and context relevance. Articles were included if their content could be verified through full-text review.

During the Full-Text Review (n=31): Articles were included if they provided sufficient information on preventive interventions for dehydration or heat stress, even if some secondary methodological details were missing. Studies were included if they reported essential demographic data (e.g., age or working conditions, among others) even if other demographic details were incomplete. Studies were retained if they presented the main findings relevant to the research question, even if some secondary statistical analyses were not reported.

During the Final Analysis (n=9): At this stage, studies were carefully evaluated to ensure they significantly contributed to addressing the research question, even if some details were incomplete. The following criteria were applied:

- **Incomplete reporting of effect size:** Studies were included if they provided sufficient information on the relationship between interventions (such as hydration, breaks, cooling) and health outcomes (e.g., reduction in dehydration or improvement in well-being), even if the full effect size was not reported.
- **Lack of specific measurement details:** Studies were retained if they clearly described their primary measures, such as the use of biomarkers or the frequency of interventions, even if some measurement details were not fully reported.

This methodological approach to managing missing data prioritized a comprehensive review while ensuring that the included studies contained sufficient information to address our research question: What dehydration prevention and management measures have been applied to agricultural workers exposed to extreme heat conditions?
