# Supplementary material for: Health Interventions for the Prevention of Dehydration in Agricultural Workers Exposed to Heat Stress: A Systematic Review
Source: Healthcare (Basel). 2025 May 23;13(11):1232. doi: 10.3390/healthcare13111232 (PMC12155329; doi:10.3390/healthcare13111232)
Supplement: Supplementary file 1 [file healthcare-13-01232-s001.zip › Supplementary material S2_new.pdf]

Supplementary material S2: FINER criteria adapted to the study

PICO	FINER	
What dehydration prevention and management measures have been applied to agricultural workers exposed to extreme heat conditions?	FEASIBLE	In the university context, it is feasible to carry out a systematic review thanks to access to databases and selected articles, in addition to having a significant population of agricultural workers, including seasonal workers and immigrants
	INTERESTING	It determines measures for the prevention and management of dehydration, providing useful evidence-based information for agricultural workers, employers, and health policy makers
	NOVEL	It offers a global perspective by synthesizing and comparing different studies and approaches on the prevention and management of dehydration in real or simulated agricultural contexts
	ETHICAL	As this systematic review does not involve the collection of primary data, approval from an ethics committee is not required. The study poses minimal ethical risks and has the potential to generate significant impact by benefiting a vulnerable population group and contributing to the development of more effective occupational health policies and practices
	RELEVANT	It improves dehydration prevention practices and could have a significant social impact on migrant seasonal workers, promoting social justice and better working conditions
