# Supplementary material for: Health Interventions for the Prevention of Dehydration in Agricultural Workers Exposed to Heat Stress: A Systematic Review
Source: Healthcare (Basel). 2025 May 23;13(11):1232. doi: 10.3390/healthcare13111232 (PMC12155329; doi:10.3390/healthcare13111232)
Supplement: Supplementary file 1 [file healthcare-13-01232-s001.zip › Supplementary material S3.pdf]

Supplementary material S3: SEARCH STRATEGY

Database	Search Strategy	Results
**PUBMED**	(("Occupational Health Services"[Mesh] OR "Preventive Health Services"[Mesh] OR "Public Health"[Mesh] OR "Organism Hydration Status"[Mesh] OR "Water-Electrolyte Balance"[Mesh]) OR "Drinking"[Mesh] OR "Fluid Therapy"[Mesh] OR (Intervention* OR strateg* OR program* OR approach* OR measure* OR techni* OR cooling OR "rest break schedul*" OR "workplace modification*" OR “workplace protection*” OR "educational campaign*" OR training OR "Preventive health" OR "Preventive program*" OR "Preventive measure*" OR prevention OR control OR "health campaign*" OR "Health Service*" OR "Health promotion" OR "Health education” OR "public health" OR “occupational Health” OR "Early Medical Intervention*"OR hydration OR Rehydration OR drink* OR cooling OR "fluid intake*" OR "water intake*" OR "water consumption" OR "fluid therap*" OR "fluid balance" OR "water electrolyte balance" OR "water-electrolyte balance")) AND (("Dehydration"[Mesh] OR "Organism Hydration Status"[Mesh] OR "Water-Electrolyte Balance"[Mesh] OR hypernatremia[Mesh] OR "Water-Electrolyte Imbalance"[Mesh]) OR (dehydration OR "fluid balance" OR "water electrolyte balance" OR "water-electrolyte balance" OR “fluid imbalance” OR "water electrolyte imbalance" OR "water-electrolyte imbalance" OR cooling OR hypernatremia OR “water loss*”)) AND (("Farmers"[Mesh] OR "Agricultural Workers' Diseases"[Mesh]) OR ("Agricultural worker*" OR "Agricultural labour force*" OR "Agricultural workforce" OR "agricultural labourer*" OR "agricultural laborer*" OR “agricultural communit*” OR "Outdoor worker*" OR "Manual worker*" OR farmer* OR farmworker* OR "farm worker*" OR “farm laborer*” OR “farm labourer*” OR "farming communit*")) AND (("Heat Stress Disorders"[Mesh] OR "Hot	67

	Temperature"[Mesh] OR "Climate Change"[Mesh] OR "Global Warming"[Mesh])<br><br>OR ("heat-related illness" OR "heat stress" OR "heat exhaustion" OR "heat stroke*" OR “heat exposure” OR "high temperature*" OR "heat wave*" OR "hot temperature*" OR “hot climate” OR "climate change" OR "global warming")) AND (“quantitative research” OR "quantitative method*" OR "quantitative stud*" OR "quantitative data" OR statistic* OR "numerical data" OR “numerical stud*” OR experiment* OR trial* OR random* OR “intervention stud*” OR quasi-experiment* OR Quasiexperimental OR “time series” OR survey* OR questionnaire* OR descriptive OR observation* OR correlation* OR interview*)	
**CINAHL**	((MH "Occupational Health Services+" OR MH "Preventive Health Care+" OR MH "Public Health+" OR MH "Hydration Status" OR MH "Fluid-Electrolyte Balance" OR MH "Fluid Intake" OR MH "Fluid Therapy+" OR MH "Beverages+")<br><br>OR (Intervention* OR strateg* OR program* OR approach* OR measure* OR techni* OR cooling OR "rest break schedul*" OR "workplace modification*" OR “workplace protection*” OR "educational campaign*" OR training OR "Preventive health" OR "Preventive program*" OR "Preventive measure*" OR prevention OR control OR "health campaign*" OR "Health Service*" OR "Health promotion" OR "Health education” OR "public health" OR “occupational Health” OR "Early Medical Intervention*"OR hydration OR Rehydration OR drinking OR cooling OR "fluid intake*" OR "water intake*" OR "water consumption" OR "fluid therap*" OR "fluid balance" OR "water electrolyte balance" OR "water-electrolyte balance")) AND ((MH "Dehydration" OR MH "Hydration Status" OR MH "Fluid-Electrolyte Balance" OR MH "Hypernatremia" OR MH "Fluid-Electrolyte Imbalancea") OR (dehydration OR "fluid balance" OR "water electrolyte balance" OR "water-electrolyte balance" OR “fluid imbalance” OR "water electrolyte imbalance" OR "water-	27

	electrolyte imbalance" OR cooling OR hypernatremia OR “water loss*”)) AND ((MH "Farmworkers") OR<br><br>("Agricultural worker*" OR "Agricultural labour force*" OR "Agricultural workforce" OR "agricultural labourer*" OR "agricultural laborer*" OR “agricultural communit*” OR "Outdoor worker*" OR "Manual worker*" OR farmer* OR farmworker* OR "farm worker*" OR “farm laborer*” OR “farm labourer*” OR "farming communit*")) AND<br><br>((MH "Heat Stress Disorders+" OR MH "Heat/Adverse Effects" OR MH "Climate Change") OR ("heat-related illness" OR "heat stress" OR "heat exhaustion" OR "heat stroke*" OR “heat exposure” OR "high temperature*" OR "heat wave*" OR "hot temperature*" OR “hot climate” OR "climate change" OR "global warming")) AND<br><br>((MH "Quantitative Studies" OR MH "Quasi-Experimental Studies+" OR MH "Experimental Studies+")<br><br>OR (“quantitative research” OR "quantitative method*" OR "quantitative stud*" OR "quantitative data" OR statistic* OR "numerical data" OR “numerical stud*” OR experiment* OR trial* OR random* OR “intervention stud*” OR quasi-experiment* OR Quasiexperimental OR “time series” OR survey* OR questionnaire* OR descriptive OR observation* OR correlation*))	
**US Department of Agriculture. National Agricultural Library**	(worker* OR "labour force*" OR workforce OR laborer* OR labourer* OR farmer* OR farmworker* OR “agricultural communit*” OR "farming communit*")<br><br>AND<br><br>("heat-related illness" OR "heat stress" OR "heat exhaustion" OR "heat stroke*" OR “heat exposure” OR "high temperature*" OR "heat wave*" OR "hot temperature*" OR “hot climate” OR "climate change" OR "global warming")	56

	AND<br><br>(dehydration OR "fluid balance" OR "water electrolyte balance" OR "water-electrolyte balance" OR “fluid imbalance” OR "water electrolyte imbalance" OR "water-electrolyte imbalance" OR hypernatremia)	
**SCOPUS**	(TITLE-ABS-KEY (intervention* OR strateg* OR program* OR approach* OR measure* OR techni* OR cooling OR "rest break schedul*" OR "workplace modification*" OR "workplace protection*" OR "educational campaign*" OR training OR "Preventive health" OR "Preventive program*" OR "Preventive measure*" OR prevention OR control OR "health campaign*" OR "Health Service*" OR "Health promotion" OR "Health education" OR "public health" OR "occupational Health" OR "Early Medical Intervention*" OR hydration OR rehydration OR drinking OR cooling OR "fluid intake*" OR "water intake*" OR "water consumption" OR "fluid therap*" OR "fluid balance" OR "water electrolyte balance" OR "water-electrolyte balance") AND TITLE-ABS-KEY (dehydration OR "fluid balance" OR "water electrolyte balance" OR "water-electrolyte balance" OR “fluid imbalance” OR "water electrolyte imbalance" OR "water-electrolyte imbalance" OR cooling OR hypernatremia OR “water loss*”) AND TITLE-ABS-KEY ("Agricultural worker*" OR "Agricultural labour force*" OR "Agricultural workforce" OR "agricultural labourer*" OR "agricultural laborer*" OR "agricultural communit*" OR "Outdoor worker*" OR "Manual worker*" OR farmer* OR farmworker* OR "farm worker*" OR "farm laborer*" OR "farm labourer*" OR "farming communit*") AND TITLE-ABS-KEY ("heat-related illness" OR "heat stress" OR "heat exhaustion" OR "heat stroke*" OR "heat exposure" OR "high temperature*" OR "heat wave*" OR "hot temperature*" OR "hot climate" OR "climate change" OR "global warming") AND TITLE-ABS-KEY ("quantitative research" OR "quantitative method*" OR "quantitative stud*" OR "quantitative data" OR statistic* OR "numerical data" OR "numerical stud*" OR experiment* OR trial* OR random* OR "intervention stud*" OR quasi-experiment* OR experimental	131

	OR "time series" OR survey* OR questionnaire* OR descriptive OR observation* OR correlation* OR interview*)	
**WoS - All databases**	TS=(intervention* OR strateg* OR program* OR approach* OR measure* OR techni* OR cooling OR "rest break schedul*" OR "workplace modification*" OR "workplace protection*" OR "educational campaign*" OR training OR "Preventive health" OR "Preventive program*" OR "Preventive measure*" OR prevention OR control OR "health campaign*" OR "Health Service*" OR "Health promotion" OR "Health education" OR "public health" OR "occupational Health" OR "Early Medical Intervention*" OR hydration OR rehydration OR drinking OR cooling OR "fluid intake*" OR "water intake*" OR "water consumption" OR "fluid therap*" OR "fluid balance" OR "water electrolyte balance" OR "water-electrolyte balance") AND TS=(dehydration OR "fluid balance" OR "water electrolyte balance" OR "water-electrolyte balance" OR “fluid imbalance” OR "water electrolyte imbalance" OR "water-electrolyte imbalance" OR cooling OR hypernatremia OR “water loss*”) AND TS=("Agricultural worker*" OR "Agricultural labour force*" OR "Agricultural workforce" OR "agricultural labourer*" OR "agricultural laborer*" OR "agricultural communit*" OR "Outdoor worker*" OR "Manual worker*" OR farmer* OR farmworker* OR "farm worker*" OR "farm laborer*" OR "farm labourer*" OR "farming communit*") AND TS=("heat-related illness" OR "heat stress" OR "heat exhaustion" OR "heat stroke*" OR "heat exposure" OR "high temperature*" OR "heat wave*" OR "hot temperature*" OR "hot climate" OR "climate change" OR "global warming") AND TS=("quantitative research" OR "quantitative method*" OR "quantitative stud*" OR "quantitative data" OR statistic* OR "numerical data" OR "numerical stud*" OR experiment* OR trial* OR random* OR "intervention stud*" OR quasi-experiment* OR experimental OR "time series" OR survey* OR questionnaire* OR descriptive OR observation* OR correlation* OR interview*)	262
**Open Access**	(intervention* OR strateg* OR program* OR approach* OR measure* OR techni* OR cooling OR "rest break schedul*" OR "workplace modification*" OR "workplace protection*" OR "educational campaign*" OR training OR "Preventive	15

**Theses and Dissertations**	health" OR "Preventive program*" OR "Preventive measure*" OR prevention OR control OR "health campaign*" OR "Health Service*" OR "Health promotion" OR "Health education" OR "public health" OR "occupational Health" OR "Early Medical Intervention*" OR hydration OR rehydration OR drinking OR cooling OR "fluid intake*" OR "water intake*" OR "water consumption" OR "fluid therap*" OR "fluid balance" OR "water electrolyte balance" OR "water-electrolyte balance") AND (dehydration OR "fluid balance" OR "water electrolyte balance" OR "water-electrolyte balance" OR “fluid imbalance” OR "water electrolyte imbalance" OR "water-electrolyte imbalance" OR cooling OR hypernatremia) AND ("Agricultural worker*" OR "Agricultural labour force*" OR "Agricultural workforce" OR "agricultural labourer*" OR "agricultural laborer*" OR "agricultural communit*" OR "Outdoor worker*" OR "Manual worker*" OR farmer* OR farmworker* OR "farm worker*" OR "farm laborer*" OR "farm labourer*" OR "farming communit*") AND ("heat-related illness" OR "heat stress" OR "heat exhaustion" OR "heat stroke*" OR "heat exposure" OR "high temperature*" OR "heat wave*" OR "hot temperature*" OR "hot climate" OR "climate change" OR "global warming") AND ("quantitative research" OR "quantitative method*" OR "quantitative stud*" OR "quantitative data" OR statistic* OR "numerical data" OR "numerical stud*" OR experiment* OR trial* OR random* OR "intervention stud*" OR quasi-experiment* OR experimental OR "time series" OR survey* OR questionnaire* OR descriptive OR observation* OR correlation*)	
	**TOTAL**	**558**
