# Supplementary material for: Health Interventions for the Prevention of Dehydration in Agricultural Workers Exposed to Heat Stress: A Systematic Review
Source: Healthcare (Basel). 2025 May 23;13(11):1232. doi: 10.3390/healthcare13111232 (PMC12155329; doi:10.3390/healthcare13111232)
Supplement: Supplementary file 1 [file healthcare-13-01232-s001.zip › Supplementary material S4.pdf]

Supplementary material S4. GRADE Assessment

Article title	Study Design – Initial Quality	Limitations of the Study	Inconsistency of the Results	Indirectness	Imprecision	Publication bias	Strong association	Consistency with the Existing Evidence	Balance of Benefits and Harms	Degree of recommendation
Mizelle 2024	Post-intervention study<br><br>YES= +1	No<br><br>PARTIAL= 0	It is not mentioned.<br><br>PARTIAL= 0	Specific population<br><br>PARTIAL= 0	Moderate sample size (47 participants)<br><br>PARTIAL= 0	Published in a peer-reviewed journal (Journal of Agromedicine)<br><br>YES= +1	It is not mentioned<br><br>PARTIAL = 0	Consistent with previous research suggesting that portable hydration systems may improve water intake and reduce the risk of heat-related illnesses<br><br>YES= +1	The benefits of improving hydration and reducing HRI appear to outweigh potential drawbacks, such as the added weight of the backpack.<br><br>YES= +1	High
Chicas 2021	Randomized pilot study.<br><br>YES= +1	Loss to follow-up<br><br>NO = -1	It is not mentioned.<br><br>PARTIAL= 0	Specific population<br><br>PARTIAL= 0	Moderate sample size (84 participants)<br><br>YES= +1	Peer-reviewed journal (Workplace Health & Safety)<br><br>YES= +1	It is not mentioned<br><br>PARTIAL = 0	Consistent with previous research suggesting that cooling devices can mitigate thermal stress<br><br>YES= +1	The potential benefits of reducing body temperature and HRI symptoms appear to outweigh the potential drawbacks.<br><br>YES= +1	Moderate
Glaser 2020	Longitudinal study with intervention	Loss to follow-up<br><br>NO = -1	It is not mentioned.<br><br>PARTIAL= 0	Specific population	Large sample size (525 in harvest 1	Peer-reviewed journal (Occupational	It is not mentioned	Consistent with previous research suggesting	The potential benefits of reducing kidney injury	Moderate.

Article title	Study Design – Initial Quality	Limitations of the Study	Inconsistency of the Results	Indirectness	Imprecision	Publication bias	Strong association	Consistency with the Existing Evidence	Balance of Benefits and Harms	Degree of recommendation
	YES= +1			PARTIAL= 0	and 567 in harvest 2)<br><br>YES= +1	and Environmental Medicine<br><br>YES= +1	PARTIAL = 0	that improving working conditions can mitigate heat stress and reduce the risk of kidney injury<br><br>YES= +1	and improving worker health appear to outweigh the potential drawbacks<br><br>YES= +1	
Luque 2020	Intervention study.<br><br>YES= +1	No<br><br>PARTIAL= 0	It is not mentioned<br><br>PARTIAL= 0	Specific population<br><br>PARTIAL= 0	Large sample size (101 participants)<br><br>YES= +1	Peer-reviewed journal (Journal of Agromedicine)<br><br>YES= +1	It is not mentioned<br><br>PARTIAL = 0	Consistent with previous research suggesting that heat safety training can mitigate the risk of HRI<br><br>YES= +1	The potential benefits of improving heat safety knowledge and practices appear to outweigh the potential drawbacks<br><br>YES= +1	High
Sorensen 2020	Intervention study<br><br>YES= +1	Loss to follow-up<br><br>NO = -1	It is not mentioned<br><br>PARTIAL= 0	Specific population<br><br>PARTIAL= 0	Large sample size (517 and 483 participants)<br><br>YES= +1	Peer-reviewed journal (International Journal of Environmental Research and Public Health)<br><br>YES= +1	It is not mentioned<br><br>PARTIAL = 0	Consistent with previous research suggesting that interventions to reduce heat stress and improve hydration may	The potential benefits of improving kidney function and reducing the risk of CKDu appear to outweigh the potential drawbacks	Moderate

Article title	Study Design – Initial Quality	Limitations of the Study	Inconsistency of the Results	Indirectness	Imprecision	Publication bias	Strong association	Consistency with the Existing Evidence	Balance of Benefits and Harms	Degree of recommendation
								mitigate renal decline<br><br>YES= +1	YES= +1	
Wegman 2018	Experimental study in a climatic chamber<br><br>YES= +1	Loss to follow-up<br><br>NO = -1	It is not mentioned<br><br>PARTIAL= 0	Specific population<br><br>PARTIAL= 0	Large sample size (117 participants)<br><br>YES= +1	Peer-reviewed journal (Scandinavian Journal of Work, Environment & Health)<br><br>YES= +1	It is not mentioned<br><br>PARTIAL = 0	Consistent with previous research suggesting that interventions to reduce heat stress and improve hydration may mitigate renal decline<br><br>YES= +1	The potential benefits of improving kidney function and reducing the risk of CKDu appear to outweigh the potential drawbacks<br><br>YES= +1	Moderate
Bodin 2016	Intervention study<br><br>YES= +1	Loss to follow-up<br><br>NO = -1	It is not mentioned<br><br>PARTIAL= 0	Specific population<br><br>PARTIAL= 0	Moderate sample size (60 participants)	Peer-reviewed journal (Occupational and Environmental Medicine)<br><br>YES= +1<br><br>YES= +1	It is not mentioned<br><br>PARTIAL = 0	Consistent with previous research suggesting that interventions to reduce heat stress and improve hydration may mitigate renal decline and improve work efficiency<br><br>YES= +1	The potential benefits of improving kidney function, reducing symptoms of heat stress, and maintaining production appear to outweigh the potential drawbacks	Moderate

Article title	Study Design – Initial Quality	Limitations of the Study	Inconsistency of the Results	Indirectness	Imprecision	Publication bias	Strong association	Consistency with the Existing Evidence	Balance of Benefits and Harms	Degree of recommendation
									YES= +1	
Choi 2008	Experimental study<br><br>YES= +1	No<br><br>PARTIAL= 0	It is not mentioned<br><br>PARTIAL= 0	Specific population<br><br>PARTIAL= 0	Small sample size (12 participants)<br><br>NO = -1	Peer-reviewed journal (Industrial Health)<br><br>YES= +1	It is not mentioned<br><br>PARTIAL = 0	Consistent with previous research suggesting that personal cooling equipment can mitigate heat stress<br><br>YES= +1	The potential benefits of reducing heat stress and improving thermal comfort appear to outweigh the potential drawbacks<br><br>YES= +1	Moderate
Hayashi 2000	Experimental study<br><br>YES= +1	No<br><br>PARTIAL= 0	It is not mentioned<br><br>PARTIAL= 0	Specific population<br><br>PARTIAL= 0	Small sample size (5 participants)<br><br>NO = -1	Peer-reviewed journal (International Archives of Occupational and Environmental Health)<br><br>YES= +1	It is not mentioned<br><br>PARTIAL = 0	Consistent with previous research suggesting that improvements in protective clothing can mitigate heat stress<br><br>YES= +1	The potential benefits of reducing heat stress and improving comfort appear to outweigh the potential drawbacks<br><br>YES= +1	Moderate
